# AMP-activated protein kinase controls exercise training- and AICAR-induced increases in SIRT3 and MnSOD

**DOI:** 10.3389/fphys.2015.00085

**Published:** 2015-03-24

**Authors:** Josef Brandauer, Marianne A. Andersen, Holti Kellezi, Steve Risis, Christian Frøsig, Sara G. Vienberg, Jonas T. Treebak

**Affiliations:** ^1^Section of Integrative Physiology, The Novo Nordisk Foundation Center for Basic Metabolic Research, University of CopenhagenCopenhagen, Denmark; ^2^Department of Health Sciences, Gettysburg CollegeGettysburg, PA, USA; ^3^Section of Molecular Physiology, Department of Nutrition, Exercise and Sports, The August Krogh Centre, University of CopenhagenCopenhagen, Denmark

**Keywords:** AMPK, SIRT3, MnSOD, SOD2, ROS, OSCP, exercise training, mitochondrial biogenesis

## Abstract

The mitochondrial protein deacetylase sirtuin (SIRT) 3 may mediate exercise training-induced increases in mitochondrial biogenesis and improvements in reactive oxygen species (ROS) handling. We determined the requirement of AMP-activated protein kinase (AMPK) for exercise training-induced increases in skeletal muscle abundance of SIRT3 and other mitochondrial proteins. Exercise training for 6.5 weeks increased SIRT3 (*p* < 0.01) and superoxide dismutase 2 (MnSOD; *p* < 0.05) protein abundance in quadriceps muscle of wild-type (WT; *n* = 13–15), but not AMPK α2 kinase dead (KD; *n* = 12–13) mice. We also observed a strong trend for increased MnSOD abundance in exercise-trained skeletal muscle of healthy humans (*p* = 0.051; *n* = 6). To further elucidate a role for AMPK in mediating these effects, we treated WT (*n* = 7–8) and AMPK α2 KD (*n* = 7–9) mice with 5-amino-1-β-D-ribofuranosyl-imidazole-4-carboxamide (AICAR). Four weeks of daily AICAR injections (500 mg/kg) resulted in AMPK-dependent increases in SIRT3 (*p* < 0.05) and MnSOD (*p* < 0.01) in WT, but not AMPK α2 KD mice. We also tested the effect of repeated AICAR treatment on mitochondrial protein levels in mice lacking the transcriptional coactivator peroxisome proliferator-activated receptor γ-coactivator 1α (PGC-1α KO; *n* = 9–10). Skeletal muscle SIRT3 and MnSOD protein abundance was reduced in sedentary PGC-1α KO mice (*p* < 0.01) and AICAR-induced increases in SIRT3 and MnSOD protein abundance was only observed in WT mice (*p* < 0.05). Finally, the acetylation status of SIRT3 target lysine residues on MnSOD (K122) or oligomycin-sensitivity conferring protein (OSCP; K139) was not altered in either mouse or human skeletal muscle in response to acute exercise. We propose an important role for AMPK in regulating mitochondrial function and ROS handling in skeletal muscle in response to exercise training.

## Introduction

Mitochondrial density and capacity for oxidative ATP synthesis in skeletal muscle are tightly linked to cellular energetic demands (Spina et al., 1996; Egan and Zierath, 2013). Protein deacetylases such as sirtuins (SIRTs) are important modulators of gene expression and protein activity and are involved in mitochondrial biogenesis. SIRT1 is mainly located in the nucleus and deacetylates the transcriptional coactivator, peroxisome proliferator-activated receptor γ-coactivator 1α (PGC-1α) (Nemoto et al., 2005), thereby increasing mitochondrial biogenesis (Puigserver et al., 1998) and improving mitochondrial function (Gerhart-Hines et al., 2007). PGC-1α also induces gene expression of the mitochondrial sirtuin SIRT3 (Schwer et al., 2002) in muscle cells and hepatocytes (Kong et al., 2010).

SIRT3 modulates mitochondrial gene expression and function. SIRT3-driven processes occur via a general deacetylation of mitochondrial proteins (Lombard et al., 2007), including key elements of the citric acid cycle and proteins involved in oxidative phosphorylation (Hallows et al., 2006; Schwer et al., 2006; Wu et al., 2013; Vassilopoulos et al., 2014), or reactive oxygen species (ROS) handling (Someya et al., 2010; Tseng et al., 2013). Thus, SIRT1 may increase SIRT3 expression via PGC-1α deacetylation to facilitate an increase in mitochondrial function and/or density.

Exercise training and caloric restriction (CR) induce mitochondrial biogenesis, but SIRT1-mediated adaptations to exercise and CR are blunted in AMP-activated protein kinase (AMPK)-deficient skeletal muscle (Cantó et al., 2010). AMPK is a heterotrimeric protein consisting of multiple catalytic (α1, α2) and regulatory (β1, β2, γ1, γ2, γ3) subunits. AMPK functions as a major sensor of cellular energy status and can be activated pharmacologically and in response to muscle contraction and CR (Koh et al., 2008). Importantly, AMPK directly phosphorylates PGC-1α (Jäger et al., 2007) which may result in SIRT1-mediated deacetylation and activation of PGC-1α (Cantó et al., 2009) and thus link cellular energy status, mitochondrial biogenesis, and ROS handling.

AMPK activation via 5-amino-1-β-D-ribofuranosyl-imidazole-4-carboxamide (AICAR) increases SIRT3 mRNA level in hepatocytes (Buler et al., 2014). While SIRT3 reduces oxidative stress induced by CR (Someya et al., 2010), AMPK partly coordinates the cellular response to CR (Shinmura et al., 2007), supporting the hypothesis that AMPK may regulate SIRT3.

Exercise training increases ROS processing in skeletal muscle (Parise et al., 2005) via a mechanism that is incompletely understood. A potential mitochondrial signaling cascade response involving SIRT3 and FOXO3A-dependent transcription of catalase and MnSOD has been proposed (Jacobs et al., 2008), but the role of this cascade in exercise-training induced adaptations is unknown. MnSOD and catalase govern the conversion of superoxide to water and oxygen in sequential steps (Reid, 2001). Activation of MnSOD is enhanced by SIRT3-dependent deacetylation at K122, enabling the cell to scavenge ROS (Tao et al., 2010). Other regulatory acetylation sites have also been reported (Qiu et al., 2010; Chen et al., 2011), but the importance for each of these sites in relation to different stimuli remains elusive.

In summary, activation of AMPK via exercise or pharmacological treatment may phosphorylate PGC-1α, increase SIRT3 protein expression, and enhance the ability of skeletal muscle to better handle ROS via deacetylation and activation of MnSOD. Whether this pathway is redundant or exclusively dependent on AMPK is unknown. Given the functional connection between AMPK activators and PGC-1α-dependent increases of SIRT3 abundance and its downstream targets, we determined the effects of exercise training and pharmacological AMPK activation via AICAR in mouse models overexpressing a kinase dead version of the catalytic α2 AMPK subunit dominant in skeletal muscle as well as in skeletal muscle of PGC-1α deficient mice.

## Materials and methods

### Exercise training—humans

Skeletal muscle samples were obtained from young male subjects previously described in an earlier study (Frøsig et al., 2004). The exercise training study was performed in compliance with the Helsinki II Declaration, with approval from the local ethics committee (#KF 01-070/96). Exercise training consisted of 15 sessions of one-legged knee extensor endurance training over the course of 3 weeks. The duration of the exercise sessions started at 1 h per session and was gradually increased to 2 h per session for the final 5 sessions. Needle biopsies were obtained under local anesthesia (2% lidocaine) from the right and left *vastus lateralis* muscles before training and 15 h (*n* = 8) after the final exercise bout.

### Acute exercise—humans

*Vastus lateralis* muscle samples were obtained from healthy young men before and after an acute bout of one-legged knee-extensor exercise. Selected data from this experiment have been published previously (Treebak et al., 2014). This study was approved by the local ethics committee (#KF 1277313) and complied with the Helsinki II Declaration. Seven subjects volunteered to perform an acute bout of one-legged knee-extensor exercise at 80% of peak work load for 1 h during the morning after an overnight fast. In the 1-h exercise bout, peak work load was increased to 100% for 5 min intervals after 15 min and again after 35 min. Needle biopsies from the right and left *vastus lateralis* muscle were obtained before (Pre) and immediately after (Post) exercise cessation as described (Treebak et al., 2014).

### Animal experiments

All animal experiments complied with the European Convention for the protection of Vertebrate Animals used for Experiments and Other Scientific Purposes (Council of Europe 123, Strasbourg, France, 1985) and were approved by the Danish Animal Experimental Inspectorate (#2012-15-2934-26 and #2012-15-2934-307).

### Exercise training—mice

Female mice (9–15 weeks of age) overexpressing a kinase dead (KD) α2 AMPK subunit in skeletal muscle (Mu et al., 2001) or WT littermates underwent 6.5 weeks of endurance exercise training (*n* = 12–15/group). Selected data from these experiments have been published previously (Brandauer et al., 2013). Mice had free access to running wheels for voluntary running 7 days/week. Running distance was recorded using a cycle odometer (#BC1009, Sigma Sport®, Germany). WT and AMPK α2 KD mice performed similar amounts of voluntary running (Brandauer et al., 2013). In addition, mice were exercised 1 h/day at 16 m/min on a motorized treadmill (Exer-3/6, Columbus Instruments) on weekdays as described (Brandauer et al., 2013). The treadmill was horizontal the first week, and the incline was increased by 2.5° per week up to 10° where it remained for the duration of the study. The morning following the final exercise bout, mice were anesthetized by an intraperitoneal (i.p.) injection using Avertin (250 mg tribromoethanol/kg body weight). Quadriceps muscles were removed, snap-frozen in liquid nitrogen, and stored at −80°C until further analyses.

### AICAR treatment studies

To determine whether AICAR-induced increases in SIRT3 and other mitochondrial proteins depend on AMPK, male AMPK α2 KD and WT mice (11–12 weeks of age) received a daily subcutaneous injection of 500 mg/kg body weight AICAR or 0.9% NaCl solution for 4 weeks (*n* = 7–8) as described (Brandauer et al., 2013). Mice were anesthetized via i.p. injection of Avertin (250 mg/kg body weight) 24 h following the last AICAR/vehicle treatment to avoid any confounding effects of the last AICAR/saline injection. Quadriceps muscle was obtained, snap-frozen in liquid N_2_, and stored at −80°C. Samples from a previously published study of male and female whole-body PGC-1α KO and WT mice were also analyzed. The mice in this study were treated with AICAR under the same conditions as described above (Leick et al., 2009, 2010a). In order to determine the acute effect of a single injection of AICAR, we analyzed samples originating from a previous study (Leick et al., 2010a). Male and female PGC-1α KO mice and WT littermates (*n* = 6–8) were injected subcutaneously with a single dose of AICAR (500 mg/kg body weight) or saline. Quadriceps muscles were taken 4 h after the injection, snap-frozen in liquid N_2_ and stored at −80°C.

### Acute exercise in previously trained or untrained mice

To assess the effects of acute exercise in trained and untrained mouse skeletal muscle, female C57BL/6JBom (Taconic, Denmark) mice (9–10 weeks of age) underwent 5.5 weeks of endurance exercise training (*n* = 26) or served as sedentary controls (*n* = 24). The training consisted of voluntary running with free access to wheel cages 7 days/week with running distance being recorded by a cycle odometer (#BC1009, Sigma Sport®, Germany). In addition, mice were exercised 1 h/day on weekdays with accelerating speeds from 8 to 18 m/min on a motorized treadmill (Exer-3/6, Columbus Instruments) or placed on a “mock” treadmill for the sedentary control group. The treadmill was horizontal the first week, and the incline was increased by 2.5° per week up to 10° at the final week to account for increased performance and to ensure a continuous training stimulus. Untrained control animals were acclimatized to treadmill running for 15–20 min with accelerating speeds from 8 to 18 m/min at a 5° incline for three consecutive days, with 1 day of rest prior to the acute exercise experiment. On the experimental day, both trained and sedentary control mice were divided into three groups: non-exercised controls (*n* = 8 sedentary, 9 trained), and mice performing either moderate-intensity (*n* = 8 sedentary, 8 trained) or high-intensity acute exercise (*n* = 7 sedentary, 8 trained), resulting in six experimental groups. Based on the recorded running distances of each mouse, mice were assigned to one of the three groups so that the average amount of training performed in the training period was similar between the three groups (i.e., 5.1 ± 0.6 km/day; mean ± SEM). Mice then performed an acute treadmill exercise bout for 1 h or served as sedentary controls. “Moderate intensity” was defined as 12 m/min at 0° incline, while “high intensity” was defined as 18 m/min with a 10° incline. Mice were euthanized by cervical dislocation immediately after the acute exercise bout. Quadriceps muscles were quickly removed, snap frozen in liquid nitrogen, and stored at –80° until further analysis.

### Tissue processing and western blot analyses

All muscle samples were processed using steel bead homogenization (Tissue Lyser II, Qiagen) in ice-cold lysis buffer (pH 7.4; 10% glycerol; 1% IGEPAL; Hepes, 50 mM; NaCl, 150 mM; NaF, 10 mM; EDTA, 1 mM; EGTA, 1 mM; sodium pyrophosphate, 20 mM; sodium orthovanadate, 2 mM; sodium-pyrophosphate 1 mM; nicotinamide 5 mM; Thiamet G 4μM and protease inhibitors (SigmaFast, Sigma Aldrich) according to manufacturer's instructions). Protein concentrations were determined using BCA assays (Thermo Scientific, #23223 and #23224). Equal amounts of protein were resolved by SDS-PAGE and transferred to polyvinylidene difluoride (PVDF) membranes (Millipore, Denmark) as described (Brandauer et al., 2013). Western blots were executed in a balanced design, with samples from all experimental conditions present on all gels, and identical internal control samples included on each blot. The internal control sample was prepared as a pool of all samples from a given experiment. All samples on individual gels were normalized to the internal control sample in order to permit comparison of samples resolved on separate gels. Following transfer, samples were subjected to immunoblot analysis to detect protein abundance using following antibodies against SIRT3 (Cell Signaling, #5490S), SIRT1 (Millipore, #07-131), MnSOD, (Millipore, #06-948), MnSOD K122 acetylation (kindly provided by Prof. David Gius, Northwestern University Feinberg School of Medicine, Chicago, Illinois; see Tao et al., 2010) and Catalase (R&D Systems, #AF3398). Protein abundance of the oxidative phosphorylation complexes was determined using antibodies against Complex I (NDUFB8, Invitrogen, #459210), Complex II (Fp subunit, Invitrogen, #459200), Complex III (Core subunit I, Invitrogen, #459140), Complex IV (Subunit I, Invitrogen, #459600), Complex V (i.e., F1F0 ATP synthase; oligomycin-sensitivity conferring protein [OSCP] subunit (Santa Cruz, #sc-74786), cytochrome C (BD Biosciences #556433) and OSCP K139 acetylation (kindly provided by Prof. David Gius). The specificity of the MnSOD K122 and OSCP K139 antibody used in this study has been verified (Tao et al., 2010; Vassilopoulos et al., 2014) and was confirmed by “split-blot” analyses, where samples prepared from the same mouse quadriceps muscle or human *vastus lateralis* muscle was resolved in three adjacent wells. After transfer to PVDF, the membrane was cut through the center well and the membrane halves were probed with the total MnSOD/OSCP or MnSOD K122/OSCP K139 antibodies, respectively. Complete alignment of the bands was confirmed (**Figure 7**).

### Quantitative polymerase chain reaction (qPCR)

qPCR was performed as described (Brandauer et al., 2013). RNA from quadriceps muscles was extracted using Trizol (Invitrogen, #15596-018) and reverse transcription was performed using iScript cDNA synthesis kit (BioRad, #170-8891). Real-time qPCR was performed using 2μl of template cDNA, 300 nM of sense and antisense oligonucleotides and SYBR Green PCR Master Mix (AH Diagnostic, #600882) in a final volume of 10 μl. Fluorescence measurements and data analysis were performed on the CFX96 real time system (BioRad). Gene expression was determined using primer sequences: SIRT3 forward primer: 5′-TACAGAAATCAGTGCCCCGA-3′ and reverse primer: 5′-GGTGGACACAAGAACTGCTG-3′; MnSOD forward primer: 5′-ACTGAAGTTCAATGGTGGGG- 3′ and reverse primer: 5′-GCTTGATAGCCTCCAGCAAC- 3′ and normalized by input cDNA (Qubit ssDNA assay kit, Invitrogen, #Q10212).

### Statistics

Statistical analyses were performed by either 2 × 2 analysis of variance (ANOVA), by 2 × 3 ANOVA, or by 2 × 2 repeated-measures ANOVA as appropriate. The Tukey test was used *post-hoc*. If statistical interactions were borderline significant (0.05 < *p* < 0.1) and the observed power was low (<0.5), individual *t*-tests were performed as noted in the description of the results. As incomplete sample sets are excluded from statistical analyses in repeated-measures ANOVA, statistical analyses performed on data from the human training study were only performed on 6 sets of samples. Statistical significance was set at *p* < 0.05. All data are reported as mean ± SEM.

## Results

### Exercise training in mice increases SIRT3 and MnSOD in an AMPK-dependent manner

To verify the potency of the exercise training program and to assess the importance of AMPK on exercise-induced mitochondrial adaptations in skeletal muscle, we performed Western blot analyses on mitochondrial Complex I through V (OSCP) and cytochrome C on samples from WT and AMPK α2 KD mice that had completed 6.5 weeks of endurance exercise training. WT and AMPK α2 KD mice performed similarly in average distance run (Brandauer et al., 2013), average duration of exercise/day (WT, 140 ± 16 min/day; KD, 168 ± 29 min/day; *p* = 0.38) and average running speed (WT, 1.39 ± 0.04 km/h; KD, 1.27 ± 0.07 km/h, *p* = 0.15).

**Figure 1:**
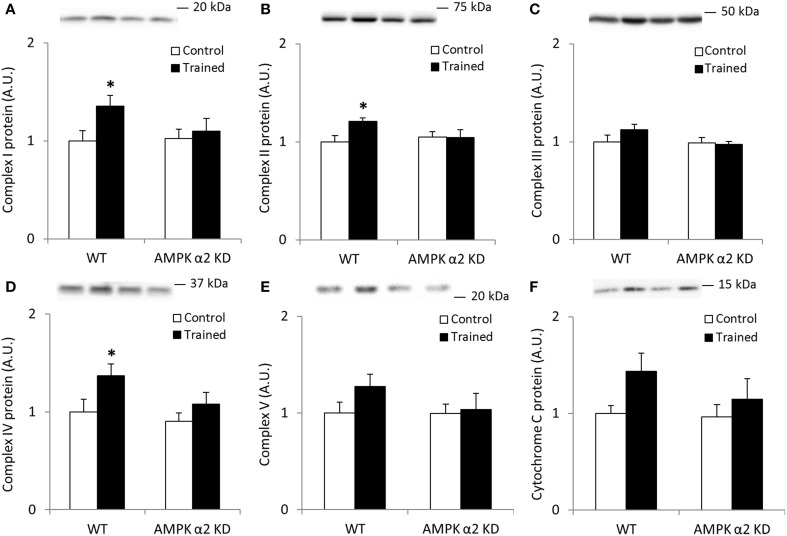
**Exercise training increase mitochondrial oxidative phosphorylation complexes in an AMPK α2-dependent manner**. Protein levels of oxidative phosphorylation complexes, **(A)** Complex I, **(B)** Complex II, **(C)** Complex III, **(D)** Complex IV, **(E)** Complex V (oligomycin-sensitivity conferring protein subunit, OSCP), and **(F)** Cytochrome C were evaluated in quadriceps muscle of sedentary (control) or exercise trained female WT and AMPK α2 KD mice (*n* = 13–15). Values are mean ± SEM. ^*^indicates vs. WT control (*p* < 0.05) analyzed by unpaired *t*-tests.

We found no interaction effects in any of the data sets presented in Figures 1A–F, likely due to low statistical power. However, if individual *t*-tests were performed between control and trained mice, we found significant (*p* < 0.05) increases in Complexes I, II, and IV protein abundance in the WT mice only. Collectively, these data suggest a regulatory role for AMPK in mediating the effects of endurance exercise training on mitochondrial protein expression.

**Figure 2:**
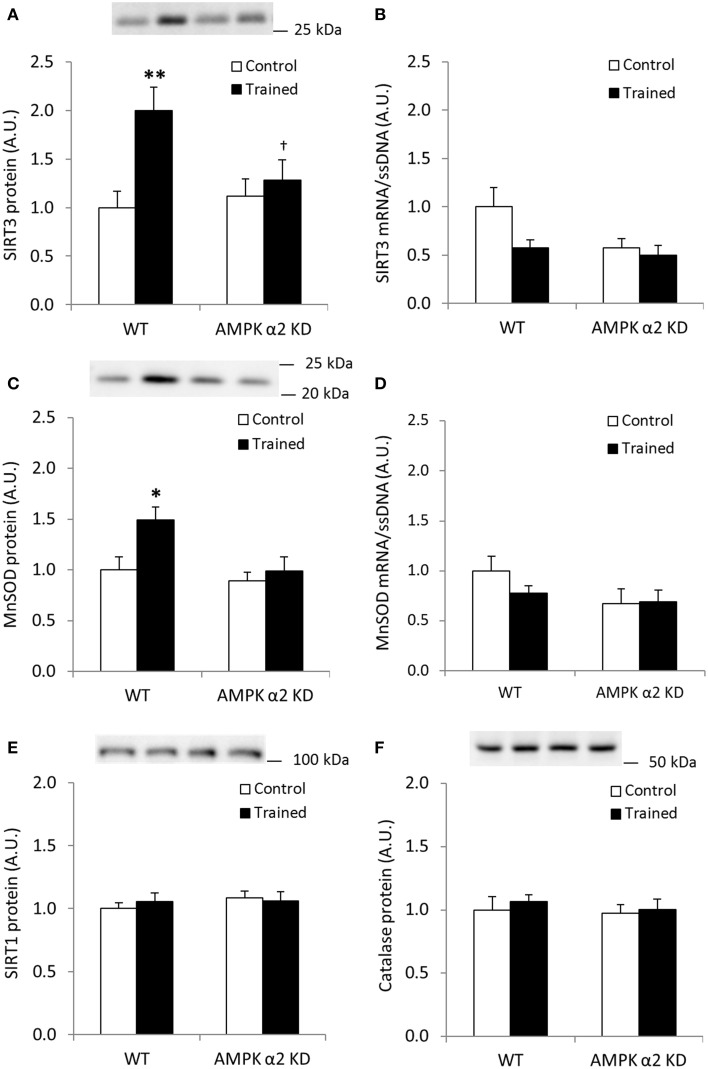
**Exercise training increases SIRT3 and MnSOD protein in mouse skeletal muscle in an AMPK α2-dependent manner. (A)** SIRT3 protein, **(B)** SIRT3 mRNA (*n* = 11–14), **(C)** MnSOD protein, **(D)** MnSOD mRNA (*n* = 11–14), **(E)** SIRT1 protein, and **(F)** Catalase protein levels were measured in quadriceps muscle of control or exercised trained female WT and AMPK α2 KD mice (*n* = 13–15 for protein measurements). Quadriceps muscles were obtained the day after the final bout of exercise. Values are mean ± SEM. An interaction effect (*p* < 0.05; treatment × genotype) was present in **(A)**. ^*^ indicates vs. WT control (*p* < 0.05; analyzed by independent *t*-test), ^**^ indicates vs. WT control (*p* < 0.01), and † indicates genotype effect within trained groups (*p* < 0.05).

Endurance exercise training increased SIRT3 protein abundance in skeletal muscle from WT, but not AMPK α2 KD mice (*p* < 0.01; *n* = 12–15) (Figure 2A). On the other hand, no statistical differences in SIRT3 mRNA levels were observed between genotypes or in response to exercise training (Figure 2B). Exercise training tended to increase skeletal muscle MnSOD protein levels in WT relative to KD mice (WT, 49%; AMPK α2 KD, 11%; genotype × treatment interaction effect, *p* = 0.079; observed power 0.294; Figure 2C). A *t*-test showed an exercise-induced increase in skeletal muscle MnSOD protein level in WT (*p* < 0.05), but not AMPK α2 KD mice. MnSOD mRNA levels were unchanged in response to exercise training in both genotypes (Figure 2D). Endurance exercise did not alter SIRT1 (Figure 2E) or catalase protein levels (Figure 2F). Thus, AMPK is required for upregulation of SIRT3 and MnSOD protein levels in response to exercise training.

### Skeletal muscle MnSOD protein abundance is increased following endurance exercise training in human skeletal muscle

**Figure 3:**
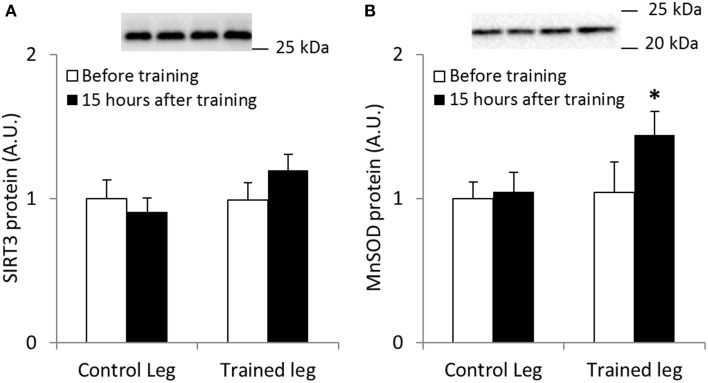
**One-legged knee-extensor endurance exercise training increases MnSOD protein levels in human *vastus lateralis* muscle**. Male individuals performed 15 sessions of 1–2 h of one-legged knee extensor endurance exercise training over a period of 3 weeks. SIRT3 and MnSOD protein levels were measured from biopsies obtained before training and 15 h after the last bout of exercise (*n* = 8). **(A)** SIRT3 and **(B)** MnSOD protein levels. A borderline interaction effect of leg × intervention (*p* = 0.051) was found for MnSOD protein levels (observed power 0.471). ^*^ indicates vs. control leg after training (*p* < 0.05; paired *t*-test). Values are mean ± SEM. Statistical analyses were performed for *n* = 6 due to incomplete sample sets.

We employed a model of one-legged endurance exercise training to study SIRT3 and MnSOD protein abundance in trained and untrained human skeletal muscle. A repeated-measures ANOVA revealed no significant changes in SIRT3 protein content following exercise training in humans (*n* = 6; Figure 3A). When samples were analyzed to determine MnSOD content, a borderline significant interaction effect (leg × intervention; *p* = 0.051, *n* = 6; observed power; 0.471; Figure 3B) was observed. A *t*-test comparing the control leg to the trained leg after the exercise intervention revealed MnSOD protein was increased in trained skeletal muscle (*p* < 0.05). Collectively, our data indicate that protein content of MnSOD is upregulated in response to exercise training in human skeletal muscle.

### Repeated AMPK activation by AICAR increases SIRT3 and MnSOD protein in an AMPK- and PGC-1α-dependent manner

**Figure 4:**
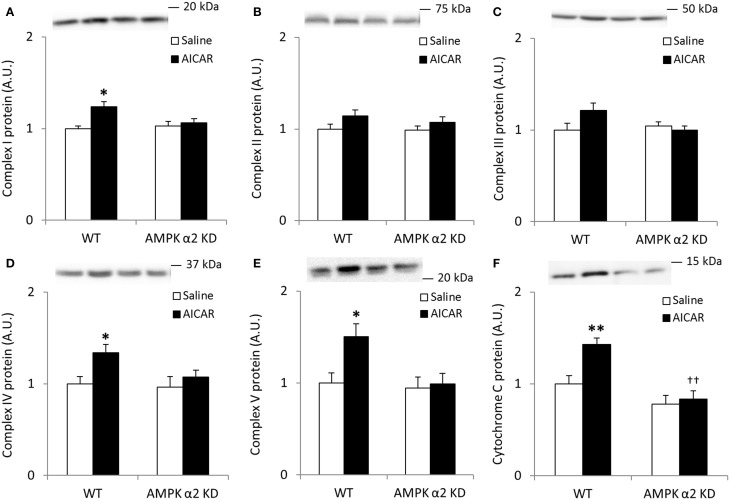
**Repeated treatment with AICAR increases abundance of mitochondrial electron transport chain proteins in an AMPK α2-dependent manner**. WT and AMPK α2 KD male mice were given daily subcutaneous injections with AICAR (500 mg/kg body weight) or saline for 4 weeks. **(A–E)** Protein abundance of oxidative phosphorylation Complexes I–V and **(F)** Cytochrome C was measured in quadriceps muscle (*n* = 7–8). A significant interaction effect (treatment × genotype; *p* < 0.05) was observed for Cytochrome C. Values are mean ± SEM. ^*^ indicates vs. WT saline (*p* < 0.05) analyzed by *t*-tests, ^**^ indicates vs. WT saline (*p* < 0.01), and †† indicates genotype effect within AICAR treated animals (*p* < 0.01).

To more directly confirm a role for AMPK in the regulation of skeletal muscle SIRT3 and MnSOD protein abundance, we treated WT and AMPK α2 KD mice with AICAR, a pharmacological AMPK activator. We performed Western blot analyses for proteins involved in oxidative phosphorylation to determine the efficacy of the AICAR treatment. Similarly to the results observed in the exercise training studies, any AICAR-induced increase in skeletal muscle abundance of oxidative phosphorylation complex proteins was blunted in AMPK α2 KD mice (Figures 4A–F). However, no significant interaction effects were found for Complex I-V, most likely due to low statistical power. When individual *t*-tests were performed, significant increases in protein content were found in the WT group for Complexes I, IV, and V. Treatment with AICAR increased abundance of cytochrome C in the WT mice (*p* < 0.01) in an AMPK-dependent manner (treatment × genotype interaction effect *p* < 0.05), and protein levels were lower in AMPK α2 KD mice (*p* < 0.01; Figure 4F).

**Figure 5:**
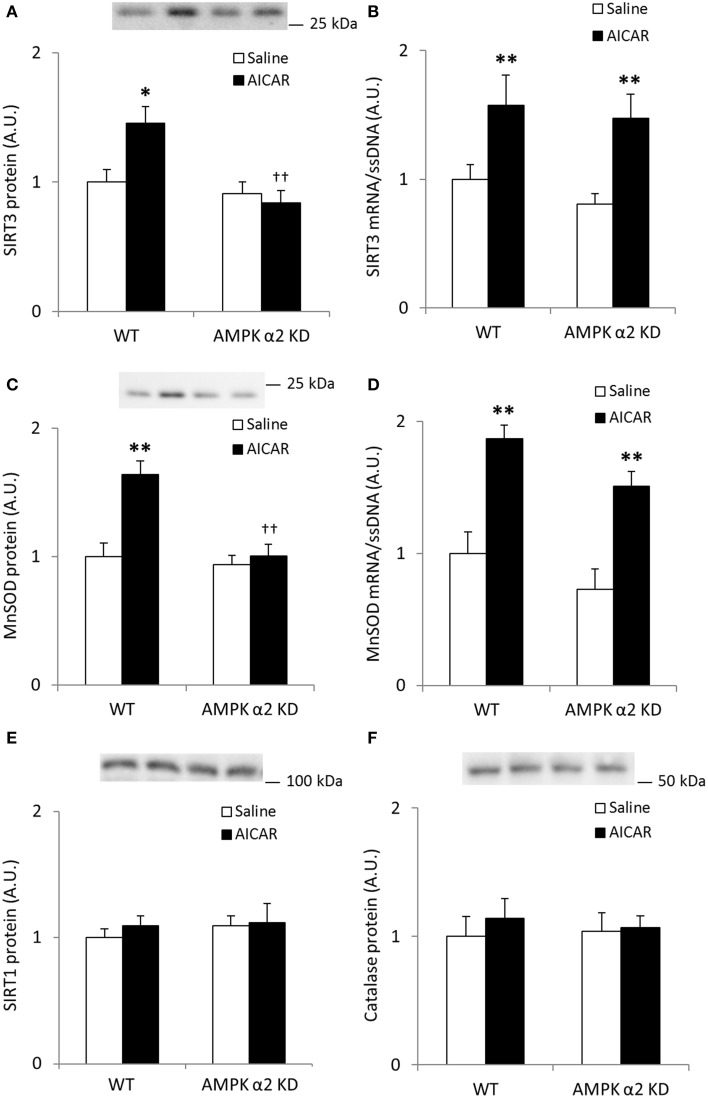
**Increases in quadriceps SIRT3 protein levels after repeated AICAR treatment are abolished in AMPK α2 KD mouse skeletal muscle**. Levels of **(A)** SIRT3 protein, **(B)** SIRT3 mRNA, **(C)** MnSOD protein, **(D)** MnSOD mRNA, **(E)** SIRT1 protein, and **(F)** Catalase protein were determined in quadriceps muscle from WT and AMPK α2 KD male mice (*n* = 7–8) treated with daily subcutaneous injections of AICAR (500 mg/kg body weight) or saline for 4 weeks. Values are mean ± SEM. Interaction effects (treatment × genotype) were present in **(A)** (*p* < 0.05) and **(C)** (*p* < 0.01). ^*^ indicates vs. saline within genotype (*p* < 0.05), ^**^ indicates vs. saline within genotype (*p* < 0.01), and †† indicates genotype effect within AICAR treated samples (*p* < 0.01).

AICAR treatment resulted in AMPK α2-dependent increases in SIRT3 protein levels (treatment × genotype interaction effect *p* < 0.05; *n* = 7–8; Figure 5A) whereas mRNA expression increased independent of genotype (*p* < 0.01; Figure 5B). Likewise, MnSOD protein levels increased in an AMPK α2-dependent manner (treatment × genotype interaction effect *p* < 0.05; *n* = 7–8; Figure 5C) while MnSOD mRNA expression increased in response to AICAR independent of AMPK α2 (*p* < 0.01, *n* = 6–8; Figure 5D). Together, these data support the hypothesis that AMPK activation governs post-transcriptional regulation of SIRT3 and MnSOD protein levels. Repeated treatment with AICAR did not increase SIRT1 or catalase protein levels (Figures 5E,F).

**Figure 6:**
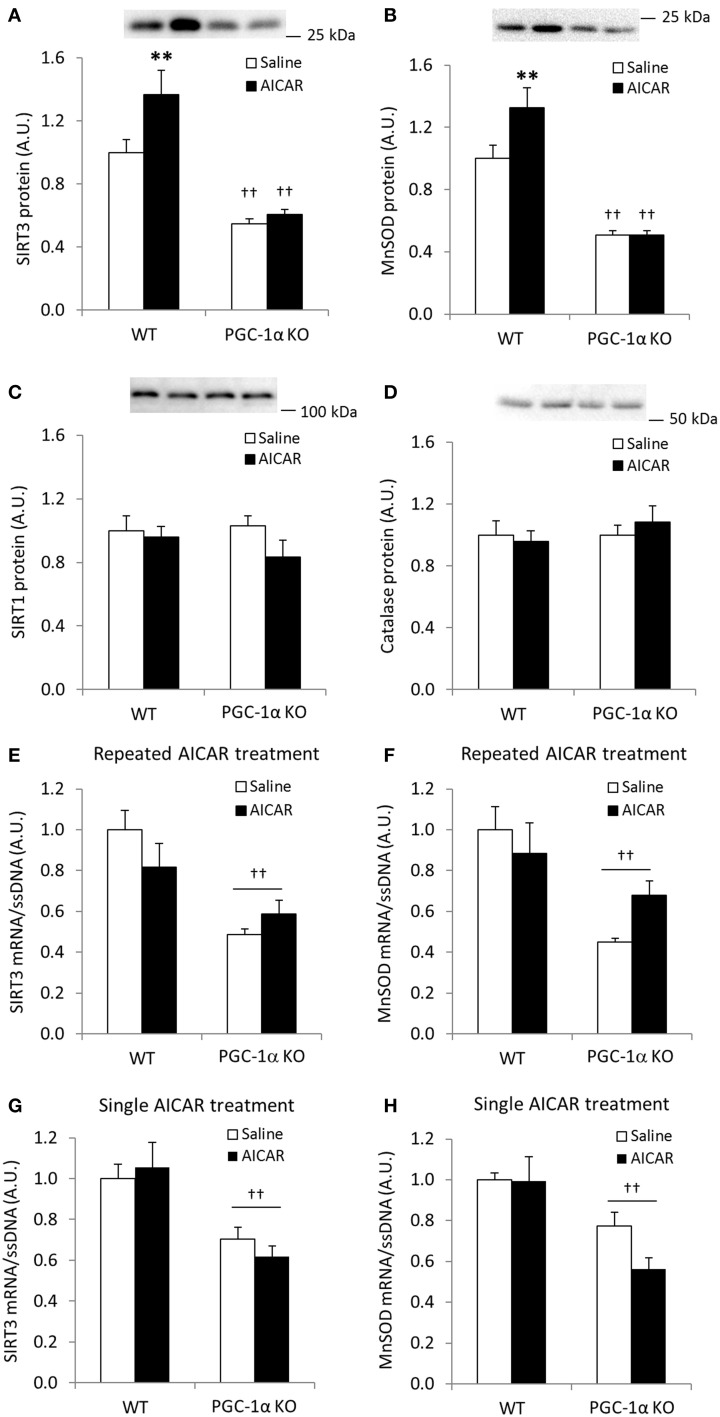
**AICAR-induced upregulation of SIRT3 and MnSOD protein in mouse skeletal muscle is dependent on PGC-1α**. WT and PGC-1α KO mice received daily subcutaneous injection of AICAR (500 mg/kg body weight) or saline for 4 weeks. Quadriceps muscles were obtained the day after the last injection. **(A)** SIRT3 protein, **(B)** MnSOD protein, **(C)** SIRT1 protein, **(D)** Catalase protein, **(E)** SIRT3 mRNA, **(F)** MnSOD mRNA (*n* = 9–10). mRNA levels of **(G)** SIRT3 and **(H)** MnSOD were obtained from quadriceps muscles from WT and PGC-1α KO mice 4 h after a single injection of saline or AICAR (500 mg/kg body weight, *n* = 6–8). Values are mean ± SEM. Interaction effects (treatment × genotype) were present for data in **(A, C)** (*p* < 0.05), ^**^ indicates vs. WT saline (*p* < 0.01), †† indicates genotype effect vs. WT (*p* < 0.01).

We next assessed whether increases in SIRT3 and MnSOD following AICAR treatment depend on functional PGC-1α signaling. AICAR treatment increased skeletal muscle protein level of SIRT3 and MnSOD in WT, but not in PGC-1α KO mice (treatment × genotype interaction effect *p* < 0.05; *n* = 9–10; Figures 6A,B), and total protein of SIRT3 and MnSOD was generally reduced in control PGC-1α KO mice (*p* < 0.01; *n* = 9–10; Figures 6A,B). SIRT1 and catalase protein levels were similar in the WT and PGC-1α mice and remained unaltered in response to repeated AICAR treatment (Figures 6C,D). These data suggest that SIRT3 and MnSOD protein abundance is regulated in a signaling axis involving both AMPK and PGC-1α. In an attempt to establish indirect evidence that upregulation of SIRT3 and MnSOD protein with AICAR is mediated through transcriptional regulation of the SIRT3 and MnSOD genes by a PGC-1α-dependent mechanism, we measured mRNA levels of SIRT3 and MnSOD in PGC-1α KO animals treated repeatedly with AICAR over 4 weeks. Although mRNA levels of both SIRT3 and MnSOD were reduced in the PGC-1α KO animals, no increase with AICAR was detected in either genotype (Figures 6E,F). To determine whether an acute AICAR injection would increase mRNA levels of SIRT3 and MnSOD, we analyzed samples from PGC-1α KO and WT mice taken 4 h after a single injection of AICAR/saline (Leick et al., 2010a). As in the repeated-AICAR experiment, PGC-1α KO mice had lower mRNA levels of SIRT3 and MnSOD, but AICAR did not increase SIRT3 or MnSOD mRNA (Figures 6G,H).

### Combined effects of endurance exercise training and acute exercise on mitochondrial protein acetylation state in mouse quadriceps muscle

**Figure 7:**
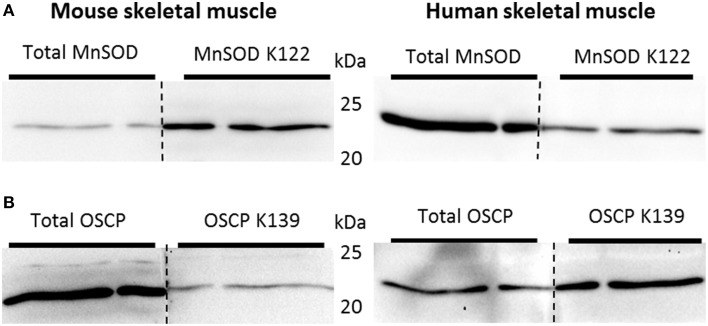
**Validation of antibodies targeting acetylated residues of MnSOD and OSCP**. Validation of antibodies targeting **(A)** acetylated residue K122 of MnSOD and total MnSOD and **(B)** OSCP acetylation at residue K139 and total OSCP in mouse and human skeletal muscle by “split-blot” analysis.

SIRT3 is involved in the activation of MnSOD by deacetylating K122. Deacetylation of this residue would be expected to occur if superoxides are produced in mitochondria during exercise. To determine whether SIRT3-mediated deacetylation of mitochondrial proteins in skeletal muscle is dependent on training status, and whether acute exercise in sedentary and exercise trained muscle would lead to a differentiated acetylation response, C57BL/6JBom mice were exercise trained or maintained as sedentary controls. On the day of tissue collection, mice were subjected to an acute 1-h bout of “moderate” or “high” intensity exercise or served as sedentary controls. Thus, using muscle samples from this experiment in addition to an antibody against acetylated K122 of MnSOD known to be important for MnSOD activity (Tao et al., 2010), the putative improved ability of trained muscle to handle ROS could be determined. To test the specificity of antibodies against total MnSOD and MnSOD K122 in skeletal muscle, we performed “split-blot” analyses and found bands from both antibodies to align in mouse and human skeletal muscle (Figure 7A). OSCP K139 antibody specificity has been previously validated (Vassilopoulos et al., 2014), and we further verified total OSCP and OSCP K139 specificity in skeletal muscle tissue by performing “split-blot” analyses and confirming that bands for OSCP and OSCP K139 align in human and mouse skeletal muscle (Figure 7B).

**Figure 8:**
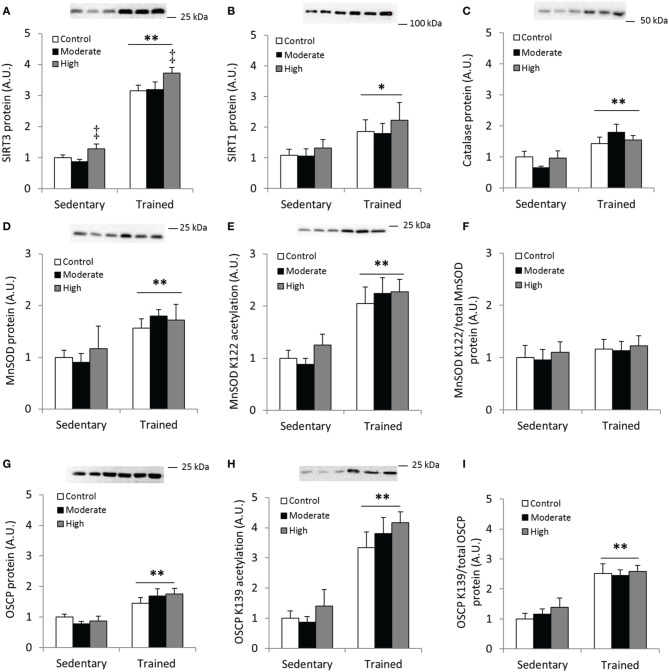
**Acute exercise does not result in deacetylation of MnSOD K122 or OSCP K139 in mouse skeletal muscle**. C57BL/6JBom mice were exercise trained for 5.5 weeks (“Trained”) or kept as sedentary controls (“Sedentary”). On the day of tissue collection, both groups were further divided into three groups that either rested (“Control”) or performed 60 min of treadmill running at either 12 m/min (0° incline; “Moderate”) or 18 m/min (10° incline; “High” intensity). **(A)** SIRT3, **(B)** SIRT1 and **(C)** Catalase protein abundance was measured in quadriceps muscles after 1 h of treadmill exercise. **(D)** Total MnSOD, **(E)** MnSOD K122 acetylation levels and **(F)** MnSOD K122 acetylation levels normalized to total MnSOD levels. **(G)** Protein levels of the Complex V subunit, F_1_F_0_ ATP synthase oligomycin-sensitivity conferring protein (OSCP), **(H)** OSCP K139 acetylation levels and **(I)** OSCP K139 acetylation/total OSCP. Values are mean ± SEM. ^*/**^ indicates main effect vs. sedentary (*p* < 0.05/0.01), ‡ main effect vs. control (*p* < 0.01), *n* = 7–9.

First, we confirmed that exercise training increases SIRT3 protein content in mouse quadriceps muscle (Figure 8A). Unexpectedly, 60 min of high-intensity acute exercise caused further increases in SIRT3 protein abundance in both previously sedentary and trained mice (Figure 8A). This increase in protein content was not accompanied by a parallel increase in SIRT3 mRNA in these samples relative to samples from any of the other groups (data not shown). Contrary to the AMPK α2 KD/WT exercise training study described above (Figures 2E,F), SIRT1 and catalase protein levels increased in response to exercise training. No additional change occurred in response to acute exercise (Figures 8B,C). Furthermore, MnSOD protein levels increased with exercise training (Figure 8D) to a similar degree as WT mice in the AMPK α2 KD/WT training study (Figure 8D vs. Figure 2C).

Although the importance of mitochondria as a source of superoxides during muscle contraction has been questioned (McArdle et al., 2004), we assessed acetylation level of MnSOD in response to exercise training and an acute bout of exercise. We detected increased acetylation of MnSOD K122 in the samples from exercise-trained animals, whereas acetylation levels were unaffected by acute exercise (Figure 8E). When MnSOD K122 acetylation status was normalized to total MnSOD protein abundance, acetylation status remained unchanged across conditions (Figure 8F).

We also tested the hypothesis that acetylation of OSCP on the K139 residue is a marker of cellular energy stress, and that exercised trained muscle would be less susceptible to exercise-induced changes in K139 acetylation. OSCP protein abundance increased with exercise training in mouse quadriceps muscle (Figure 8G), in addition to a pronounced increase in acetylation of OSCP K139 (Figure 8H). When OSCP acetylation level was normalized to total OSCP protein, K139 acetylation increased with exercise training (Figure 8I). Contrary to our hypothesis, OSCP K139 acetylation relative to total OSCP was unaffected by acute exercise (Figures 8H,I).

### Acetylation patterns in human *vastus lateralis* with acute exercise

**Figure 9:**
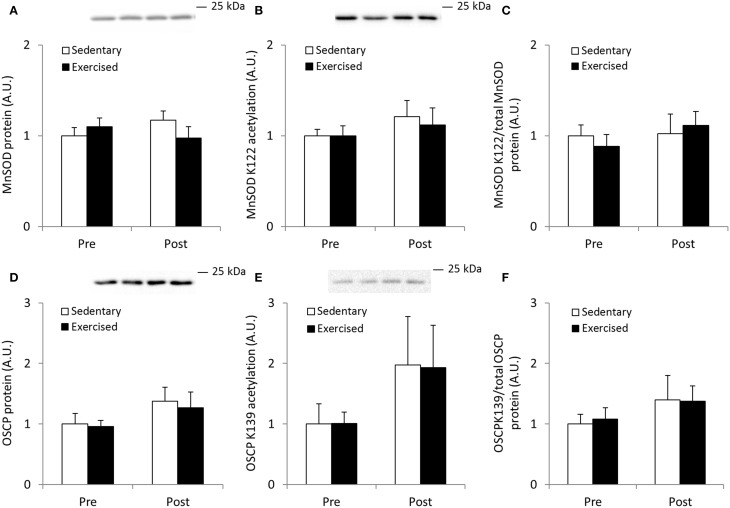
**Acetylation status of MnSOD K122 and OSCP K139 is unaffected by acute exercise in human skeletal muscle**. Male subjects performed an acute bout (1 h) of one-legged extensor exercise at 80% peak work load after an over-night fast and *vastus lateralis* muscle biopsies from both legs were obtained before (Pre) and immediately after (Post) the exercise bout (*n* = 7). **(A)** MnSOD protein, **(B)** MnSOD K122 acetylation and **(C)** MnSOD K122 acetylation/MnSOD total protein abundance. **(D)** OSCP protein, **(E)** Acetylation status of OSCP K139 and **(F)** OSCP K139 normalized to total OSCP protein abundance. Values are mean ± SEM. No significant effects were observed.

To compare the MnSOD and OSCP acetylation response obtained in exercised mouse quadriceps muscle with acutely exercised human skeletal muscle, we determined the acetylation level of these proteins in samples obtained from strenuously exercised *vastus lateralis* muscle of healthy young men. Acute exercise did not alter total protein abundance of either MnSOD or OSCP, nor did this intervention alter relative protein acetylation directly following exercise cessation (Figures 9A–F).

## Discussion

AMPK is a cellular “fuel gauge” that integrates and communicates disruptions in cellular energy charge to downstream targets (Hardie, 2011). One key signaling pathway by which AMPK exerts its effects is via the AMPK-PGC-1α-SIRT3 axis, which likely affects mitochondrial function and gene expression to adapt to metabolic changes (Hallows et al., 2006; Schwer et al., 2006; Someya et al., 2010; Tseng et al., 2013; Wu et al., 2013; Vassilopoulos et al., 2014). Here, we present evidence that AMPK is required for the increase in skeletal muscle SIRT3 and MnSOD protein abundance in addition to proteins in the mitochondrial respiratory complexes following exercise training and repeated AICAR treatment.

AMPK plays a critical role in mediating AICAR-induced increases in mitochondrial protein abundance (Jùrgensen et al., 2007). The present study confirms these findings and provides additional evidence that AMPK is required to fully beget exercise training-induced increases in mitochondrial oxidative phosphorylation complexes. This observation is in line with recent data showing that exercise training-induced adaptations in skeletal muscle on mitochondrial Complexes I-V are dependent on the upstream AMPK kinase, LKB1 (Tanner et al., 2013). Other studies employing the same (Abbott and Turcotte, 2014) or similar mouse models of AMPK deficiency (Röckl et al., 2007) as in the present investigation did not detect AMPK-dependent defects in the ability to increase mitochondrial protein content. However, these studies only assessed citrate synthase activity and did not report the abundance of electron transport chain proteins.

The one-legged endurance exercise training model represents a well-controlled method to study contraction-mediated adaptations in *vastus lateralis* muscle in humans (Andersen et al., 1985; Frøsig et al., 2004). Despite a relatively small sample size, we found near-significant increases in skeletal muscle MnSOD protein level in the trained, but not untrained leg of healthy volunteers. Conversely, SIRT3 protein levels were not increased. This is in conflict with emerging evidence that SIRT3 expression is increased in exercise-trained human and rodent skeletal muscle (Lanza et al., 2008; Palacios et al., 2009). While an early cross-sectional study reported higher protein activity of MnSOD in skeletal muscle of individuals with high aerobic fitness (Jenkins et al., 1984), some longitudinal studies have called these findings into question (Tiidus et al., 1996; Tonkonogi et al., 2000).

In our study, SIRT3 or ROS defense protein abundance was unaltered by exercise training in AMPK α2 KD mice. These results are inconsistent with the notion that SIRT3 may be induced by exercise in an AMPK-independent manner (Gurd et al., 2012). While 7 days of chronic electrical stimulation increased SIRT3 level in rat hind limb muscles, AICAR treatment had no effect (Gurd et al., 2012). Apart from obvious species and protocol differences between the two studies, these findings could also suggest additional, possibly time-sensitive, regulatory mechanisms of SIRT3 protein levels.

Activation of SIRT1 via deletion of poly (ADP-ribose) polymerase-1 (PARP-1), a major NAD-consuming enzyme, increases mitochondrial content (Bai et al., 2011). In the present study, SIRT1 and catalase protein abundance was not consistently increased with exercise training, despite a substantive increase in skeletal muscle SIRT3 protein levels in WT mice. These findings corroborate earlier reports showing that SIRT1 expression is not strongly correlated with contraction-induced changes in mitochondrial protein abundance (Chabi et al., 2009; Ringholm et al., 2013). A proportionally larger increase in SIRT1 activity, rather than protein abundance, may explain the enhanced response to endurance training and AICAR in WT mice in our study. For example, high-intensity interval training increases overall SIRT1 activity, despite decreasing SIRT1 protein concentration in humans (Gurd et al., 2010). However, SIRT1 is an NAD-dependent sirtuin, and we have previously reported that AMPK α2 KD, PGC-1α KO, and corresponding WT littermates respond similarly in increasing protein abundance of nicotinamide phosphoribosyltransferase, the rate-limiting enzyme in NAD recycling, in response to exercise training (Brandauer et al., 2013). These data suggest that the differential response in mitochondrial protein abundance following exercise training is unrelated to SIRT1 activation via NAD. Interestingly, PARP-1 deletion leads to an activation of SIRT1, but not SIRT3, suggesting possible differences in sirtuin activation in different cellular compartments (Bai et al., 2011).

While SIRT3 and MnSOD protein abundance increased in response to exercise training in WT mice, SIRT3, and MnSOD mRNA levels were unaffected in both genotypes. On the other hand, repeated AICAR treatment increased SIRT3 and MnSOD protein levels in an AMPK α2-dependent manner, while mRNA levels of SIRT3 and MnSOD were increased in both WT and AMPK α2 KD mice. These data strongly suggest that AMPK is involved in post-transcriptional regulation of SIRT3 and MnSOD gene products with AICAR. Although previous studies have failed to demonstrate a role for AMPK α2 in regulating gene expression in mouse skeletal muscle after acute exercise (Jùrgensen et al., 2005; Brandauer et al., 2013), we cannot rule out that AMPK could be important for promoting gene transcription of SIRT3 and MnSOD after exercise. Further studies are needed to clarify the differential effects of AICAR and exercise training on mRNA expression of these two genes.

We assessed the necessity of PGC-1α in mediating AICAR-induced increases in SIRT3 and MnSOD protein levels. While SIRT3 and MnSOD mRNA levels did not increase in WT or whole-body PGC-1α KO mice with either a single dose of AICAR or in response to repeated AICAR treatment, SIRT3 and MnSOD protein abundance with repeated AICAR treatment increased in WT mice but was abolished in PGC-1α KO mice. These mice also had drastically reduced SIRT3 and MnSOD protein abundance in untreated muscle, further underscoring the importance of PGC-1α in the maintenance of mitochondrial integrity. Although no data from exercise-trained PGC-1α KO muscle are presented here, exercise training restores muscle MnSOD concentration in a PGC-1α-independent manner in young mice (Geng et al., 2010), whereas the exercise training response on MnSOD protein levels has been found to be PGC-1α-dependent in old mice (Leick et al., 2010b; Olesen et al., 2013).

Our data on mitochondrial protein abundance should be interpreted in context with previously published literature on fiber type switching following exercise training or AICAR administration. AMPK and PGC-1α are vital regulators of cellular metabolic adaptations, and a functional AMPK α2 subunit appears to be required to fully realize training-induced IIb to IIa/x fiber type conversion (Röckl et al., 2007). Repeated AICAR treatment alone may be insufficient to change skeletal muscle fiber type (Bamford et al., 2003; Putman et al., 2003), although data from dystrophic mice indicates that AICAR may cause a phenotypic shift toward a more slow-twitch, oxidative fiber type under some circumstances (Ljubicic et al., 2011). PGC-1α is not only a major stimulator of mitochondrial biogenesis but also promotes type II to type I fiber type conversion (Lee et al., 2006), along with other factors such as calcineurin (Naya et al., 2000). Our data and these lines of evidence further support the notion that AMPK and PGC-1α are centrally important regulators of metabolic and contractile performance of mammalian skeletal muscle. However, the finding that AICAR treatment increases mitochondrial biogenesis, but may not necessarily change fiber type suggests that these processes may commonly occur in tandem but not be regulated by the same physiological stimuli.

SIRT3 is known to have deacetylase activity in the mitochondrion (Lombard et al., 2007). MnSOD activity is regulated through deacetylation via SIRT3 and plays an important role in handling and regulating ROS levels in mitochondria (Ahn et al., 2008; Tao et al., 2010). MnSOD has multiple acetylation sites (Rardin et al., 2013) where key lysine residues (e.g., K68 and K122) are deacetylated in response to exercise and cellular stress (Tao et al., 2010). To elucidate the interplay between aerobic fitness and stress induced by acute exercise, trained or untrained WT mice were subjected to an acute bout of “moderate” or “high”-intensity exercise, or assigned to a non-exercise control group. Despite the substantial increase in SIRT3 protein abundance, acetylation of regulatory lysine residue K122 of MnSOD or K139 of OSCP was not reduced in exercise-trained and/or acutely exercised mice at the various exercise intensities. Rather, when normalized to total protein levels, MnSOD K122 acetylation was unaltered between exercise trained and untrained mice, whereas acetylation status of K139 on OSCP was increased. This exercise training-induced increase in MnSOD implicates an enhanced cellular ROS handling capacity. However, our data on MnSOD and OSCP acetylation following acute exercise are not in obvious agreement with reported exercise-induced reductions in acetylation of these sites (Vassilopoulos et al., 2014). One difference between the two exercise protocols is that all mice in the present study completed the “moderate” and “high” intensity exercise bouts, while the exercise protocol in the previous study (Vassilopoulos et al., 2014) was exhaustive. Exhaustive exercise may be required to detect an appreciable deacetylation of MnSOD and OSCP at these lysine residues. The lack of exercise-induced changes in MnSOD and OSCP acetylation was compared with human skeletal muscle samples obtained in conjunction with a one-legged exercise protocol. Under these conditions, we also failed to observe a differential regulation of MnSOD K122 and OSCP K139 between the exercised and sedentary legs. Thus, the effects of acute exercise on MnSOD and OSCP deacetylation may vary across different experimental models and organisms. Further studies are warranted to determine the exact factors regulating SIRT3-regulated protein deacetylation with acute exercise.

The substantial increase in K139 acetylation we found after exercise training in mice may seem counterintuitive. One explanation for this observed increased OSCP acetylation could be that total cellular mitochondrial content is enhanced. Exercise training may thus have resulted in an ATP-generating potential that surpasses the actual need for ATP, leading to ATP synthase inhibition via OSCP acetylation. Another possibility is that lysine residues other than K139 on OSCP or lysines on other subunits of the ATP synthase are important for ATP synthase activity, and that acetylation status of these is changed in response to training.

The fact that concentrations of the mitochondrial SIRT3 protein were more than 3-fold higher in exercise-trained compared with untrained mice presents an intriguing observation. Why increased deacetylase protein concentration would result in unchanged or even increased acetylation levels in MnSOD or OSCP, respectively, is not immediately clear. In this context, a previous study reported that SIRT3 reduces mitochondrial protein synthesis via deacetylation of the ribosomal protein MRPL10 (Yang et al., 2010). Such an adaptation would induce an ATP-“sparing” effect that would preserve ATP pools for cross-bridge cycling and maintaining calcium homeostasis. In short, an acute increase in SIRT3 protein or activity is consistent with an inhibition of protein synthesis and other energetically costly processes by AMPK activation (Jensen et al., 2009; Hardie, 2011; White and Schenk, 2012).

In conclusion, we provide evidence for both AMPK and PGC-1α in regulating protein abundance of SIRT3 and MnSOD. These proteins are important for the regulation of mitochondrial adaptation and the handling of ROS, respectively. The interactive effects of acute and chronic exercise on MnSOD and OSCP acetylation status constitute an unexplored avenue, with implications for intensity- and duration-dependent mitochondrial adaptations that warrant further investigation.

## Author contributions

Experiments and analyses of samples in the present manuscript were conducted in Section of Integrated Physiology at the Novo Nordisk Foundation Center for Basic Metabolic Research, University of Copenhagen.

1. Conception and design of the experiments: JB, MAA, SGV, JTT.
2. Collection, analysis and interpretation of data: JB, MAA, HK, SR, CF, SGV, JTT.
3. Drafting the manuscript: JB, MAA, JTT.
4. Edited, revised and approved the final version of the manuscript: All.

## Financial support

Support for this study was provided by the Novo Nordisk Foundation Center for Basic Metabolic Research and the Novo Nordisk Foundation (Excellence Project Award) to JTT. JB was supported by a Research and Professional Development Grant by Gettysburg College.

### Conflict of interest statement

The authors declare that the research was conducted in the absence of any commercial or financial relationships that could be construed as a potential conflict of interest.

## References

[B1] Abbott M. J. Turcotte L. P. (2014). AMPK-α2 is involved in exercise training-induced adaptations in insulin-stimulated metabolism in skeletal muscle following high-fat diet. J. Appl. Physiol. 117, 869–879. 10.1152/japplphysiol.01380.2013 25103967

[B2] Ahn B.-H. Kim H.-S. Song S. Lee I. H. Liu J. Vassilopoulos A.. (2008). A role for the mitochondrial deacetylase Sirt3 in regulating energy homeostasis. Proc. Natl. Acad. Sci. U.S.A. 105, 14447–14452. 10.1073/pnas.0803790105 18794531 PMC2567183

[B3] Andersen P. Adams R. P. Sjogaard G. Thorboe A. Saltin B. (1985). Dynamic knee extension as model for study of isolated exercising muscle in humans. J. Appl. Physiol. 59, 1647–1653. 4066596 10.1152/jappl.1985.59.5.1647

[B4] Bai P. Cantó C. Oudart H. Brunyánszki A. Cen Y. Thomas C.. (2011). PARP-1 inhibition increases mitochondrial metabolism through SIRT1 activation. Cell Metab. 13, 461–468. 10.1016/j.cmet.2011.03.004 21459330 PMC3086520

[B5] Bamford J. A. Lopaschuk G. D. MacLean I. M. Reinhart M. L. Dixon W. T. Putman C. T. (2003). Effects of chronic AICAR administration on the metabolic and contractile phenotypes of rat slow- and fast-twitch skeletal muscles. Can. J. Physiol. Pharmacol. 81, 1072–1082. 10.1139/y03-110 14719043

[B6] Brandauer J. Vienberg S. G. Andersen M. A. Ringholm S. Risis S. Larsen P. S.. (2013). AMP-activated protein kinase regulates nicotinamide phosphoribosyl transferase expression in skeletal muscle. J. Physiol. 591(Pt 20), 5207–5220. 10.1113/jphysiol.2013.259515 23918774 PMC3810819

[B7] Buler M. Aatsinki S.-M. Izzi V. Uusimaa J. Hakkola J. (2014). SIRT5 is under the control of PGC-1α and AMPK and is involved in regulation of mitochondrial energy metabolism. FASEB J. 28, 3225–3237. 10.1096/fj.13-245241 24687991

[B8] Cantó C. Gerhart-Hines Z. Feige J. N. Lagouge M. Noriega L. Milne J. C.. (2009). AMPK regulates energy expenditure by modulating NAD+ metabolism and SIRT1 activity. Nature 458, 1056–1060. 10.1038/nature07813 19262508 PMC3616311

[B9] Cantó C. Jiang L. Q. Deshmukh A. S. Mataki C. Coste A. Lagouge M.. (2010). Interdependence of AMPK and SIRT1 for metabolic adaptation to fasting and exercise in skeletal muscle. Cell Metab. 11, 213–219. 10.1016/j.cmet.2010.02.006 20197054 PMC3616265

[B10] Chabi B. Adhihetty P. J. O'Leary M. F. N. Menzies K. J. Hood D. A. (2009). Relationship between Sirt1 expression and mitochondrial proteins during conditions of chronic muscle use and disuse. J. Appl. Physiol. 107, 1730–1735. 10.1152/japplphysiol.91451.2008 19797682

[B11] Chen Y. Zhang J. Lin Y. Lei Q. Guan K.-L. Zhao S.. (2011). Tumour suppressor SIRT3 deacetylates and activates manganese superoxide dismutase to scavenge ROS. EMBO Rep. 12, 534–541. 10.1038/embor.2011.65 21566644 PMC3128277

[B12] Egan B. Zierath J. R. (2013). Exercise metabolism and the molecular regulation of skeletal muscle adaptation. Cell Metab. 17, 162–184. 10.1016/j.cmet.2012.12.012 23395166

[B13] Frøsig C. Jørgensen S. B. Hardie D. G. Richter E. A. Wojtaszewski J. F. P. (2004). 5′-AMP-activated protein kinase activity and protein expression are regulated by endurance training in human skeletal muscle. Am. J. Physiol. Endocrinol. Metab. 286, E411–E417. 10.1152/ajpendo.00317.2003 14613924

[B14] Geng T. Li P. Okutsu M. Yin X. Kwek J. Zhang M.. (2010). PGC-1alpha plays a functional role in exercise-induced mitochondrial biogenesis and angiogenesis but not fiber-type transformation in mouse skeletal muscle. Am. J. Physiol. Cell Physiol. 298, C572–C579. 10.1152/ajpcell.00481.2009 20032509 PMC3353735

[B15] Gerhart-Hines Z. Rodgers J. T. Bare O. Lerin C. Kim S.-H. Mostoslavsky R.. (2007). Metabolic control of muscle mitochondrial function and fatty acid oxidation through SIRT1/PGC-1alpha. EMBO J. 26, 1913–1923. 10.1038/sj.emboj.7601633 17347648 PMC1847661

[B16] Gurd B. J. Holloway G. P. Yoshida Y. Bonen A. (2012). In mammalian muscle, SIRT3 is present in mitochondria and not in the nucleus; and SIRT3 is upregulated by chronic muscle contraction in an adenosine monophosphate-activated protein kinase-independent manner. Metab. Clin. Exp. 61, 733–741. 10.1016/j.metabol.2011.09.016 22078938

[B17] Gurd B. J. Perry C. G. R. Heigenhauser G. J. F. Spriet L. L. Bonen A. (2010). High-intensity interval training increases SIRT1 activity in human skeletal muscle. Appl. Physiol. Nutr. Metab. 35, 350–357. 10.1139/H10-030 20555380

[B18] Hallows W. C. Lee S. Denu J. M. (2006). Sirtuins deacetylate and activate mammalian acetyl-CoA synthetases. Proc. Natl. Acad. Sci. U.S.A. 103, 10230–10235. 10.1073/pnas.0604392103 16790548 PMC1480596

[B19] Hardie D. G. (2011). Energy sensing by the AMP-activated protein kinase and its effects on muscle metabolism. Proc. Nutr. Soc. 70, 92–99. 10.1017/S0029665110003915 21067629

[B20] Jacobs K. M. Pennington J. D. Bisht K. S. Aykin-Burns N. Kim H.-S. Mishra M.. (2008). SIRT3 interacts with the daf-16 homolog FOXO3a in the mitochondria, as well as increases FOXO3a dependent gene expression. Int. J. Biol. Sci. 4, 291–299. 18781224 10.7150/ijbs.4.291 PMC2532794

[B21] Jäger S. Handschin C. St-Pierre J. Spiegelman B. M. (2007). AMP-activated protein kinase (AMPK) action in skeletal muscle via direct phosphorylation of PGC-1alpha. Proc. Natl. Acad. Sci. U.S.A. 104, 12017–12022. 10.1073/pnas.0705070104 17609368 PMC1924552

[B22] Jenkins R. R. Friedland R. Howald H. (1984). The relationship of oxygen uptake to superoxide dismutase and catalase activity in human skeletal muscle. Int. J. Sports Med. 5, 11–14. 10.1055/s-2008-1025872 6607896

[B23] Jensen T. E. Wojtaszewski J. F. P. Richter E. A. (2009). AMP-activated protein kinase in contraction regulation of skeletal muscle metabolism: necessary and/or sufficient? Acta Physiol. (Oxf). 196, 155–174. 10.1111/j.1748-1716.2009.01979.x 19243572

[B24] Jørgensen S. B. Treebak J. T. Viollet B. Schjerling P. Vaulont S. Wojtaszewski J. F. P.. (2007). Role of AMPKalpha2 in basal, training-, and AICAR-induced GLUT4, hexokinase II, and mitochondrial protein expression in mouse muscle. Am. J. Physiol. Endocrinol. Metab. 292, E331–E339. 10.1152/ajpendo.00243.2006 16954334

[B25] Jørgensen S. B. Wojtaszewski J. F. P. Viollet B. Andreelli F. Birk J. B. Hellsten Y.. (2005). Effects of alpha-AMPK knockout on exercise-induced gene activation in mouse skeletal muscle. FASEB J. 19, 1146–1148. 10.1096/fj.04-3144fje 15878932

[B26] Koh H.-J. Brandauer J. Goodyear L. J. (2008). LKB1 and AMPK and the regulation of skeletal muscle metabolism. Curr. Opin. Clin. Nutr. Metab. Care 11, 227–232. 10.1097/MCO.0b013e3282fb7b76 18403917 PMC2887290

[B27] Kong X. Wang R. Xue Y. Liu X. Zhang H. Chen Y.. (2010). Sirtuin 3, a new target of PGC-1alpha, plays an important role in the suppression of ROS and mitochondrial biogenesis. PLoS ONE 5:e11707. 10.1371/journal.pone.0011707 20661474 PMC2908542

[B28] Lanza I. R. Short D. K. Short K. R. Raghavakaimal S. Basu R. Joyner M. J.. (2008). Endurance exercise as a countermeasure for aging. Diabetes 57, 2933–2942. 10.2337/db08-0349 18716044 PMC2570389

[B29] Lee W. J. Kim M. Park H.-S. Kim H. S. Jeon M. J. Oh K. S.. (2006). AMPK activation increases fatty acid oxidation in skeletal muscle by activating PPARalpha and PGC-1. Biochem. Biophys. Res. Commun. 340, 291–295. 10.1016/j.bbrc.2005.12.011 16364253

[B30] Leick L. Fentz J. Biensø R. S. Knudsen J. G. Jeppesen J. Kiens B.. (2010a). PGC-1{alpha} is required for AICAR-induced expression of GLUT4 and mitochondrial proteins in mouse skeletal muscle. Am. J. Physiol. Endocrinol. Metab. 299, E456–E465. 10.1152/ajpendo.00648.2009 20628026

[B31] Leick L. Hellsten Y. Fentz J. Lyngby S. S. Wojtaszewski J. F. P. Hidalgo J.. (2009). PGC-1alpha mediates exercise-induced skeletal muscle VEGF expression in mice. Am. J. Physiol. Endocrinol. Metab. 297, E92–E103. 10.1152/ajpendo.00076.2009 19401459

[B32] Leick L. Lyngby S. S. Wojtaszewski J. F. P. Wojtasewski J. F. P. Pilegaard H. (2010b). PGC-1alpha is required for training-induced prevention of age-associated decline in mitochondrial enzymes in mouse skeletal muscle. Exp. Gerontol. 45, 336–342. 10.1016/j.exger.2010.01.011 20085804

[B33] Ljubicic V. Miura P. Burt M. Boudreault L. Khogali S. Lunde J. A.. (2011). Chronic AMPK activation evokes the slow, oxidative myogenic program and triggers beneficial adaptations in mdx mouse skeletal muscle. Hum. Mol. Genet. 20, 3478–3493. 10.1093/hmg/ddr265 21659335

[B34] Lombard D. B. Alt F. W. Cheng H.-L. Bunkenborg J. Streeper R. S. Mostoslavsky R.. (2007). Mammalian Sir2 homolog SIRT3 regulates global mitochondrial lysine acetylation. Mol. Cell. Biol. 27, 8807–8814. 10.1128/MCB.01636-07 17923681 PMC2169418

[B35] McArdle A. van der Meulen J. Close G. L. Pattwell D. Van Remmen H. Huang T. T.. (2004). Role of mitochondrial superoxide dismutase in contraction-induced generation of reactive oxygen species in skeletal muscle extracellular space. Am. J. Physiol. Cell Physiol. 286, C1152–C1158. 10.1152/ajpcell.00322.2003 15075214

[B36] Mu J. Brozinick J. T. Jr. Valladares O. Bucan M. Birnbaum M. J. (2001). A role for AMP-activated protein kinase in contraction- and hypoxia-regulated glucose transport in skeletal muscle. Mol. Cell 7, 1085–1094. 10.1016/S1097-2765(01)00251-9 11389854

[B37] Naya F. J. Mercer B. Shelton J. Richardson J. A. Williams R. S. Olson E. N. (2000). Stimulation of slow skeletal muscle fiber gene expression by calcineurin *in vivo*. J. Biol. Chem. 275, 4545–4548. 10.1074/jbc.275.7.4545 10671477

[B38] Nemoto S. Fergusson M. M. Finkel T. (2005). SIRT1 functionally interacts with the metabolic regulator and transcriptional coactivator PGC-1{alpha}. J. Biol. Chem. 280, 16456–16460. 10.1074/jbc.M501485200 15716268

[B39] Olesen J. Ringholm S. Nielsen M. M. Brandt C. T. Pedersen J. T. Halling J. F.. (2013). Role of PGC-1α in exercise training- and resveratrol-induced prevention of age-associated inflammation. Exp. Gerontol. 48, 1274–1284. 10.1016/j.exger.2013.07.015 23916840 PMC4045249

[B40] Palacios O. M. Carmona J. J. Michan S. Chen K. Y. Manabe Y. J. L. W. III. Goodyear L. J.. (2009). Diet and exercise signals regulate SIRT3 and activate AMPK and PGC-1α in skeletal muscle. Aging (Albany. NY) 1, 771–783. 20157566 10.18632/aging.100075 PMC2815736

[B41] Parise G. Phillips S. M. Kaczor J. J. Tarnopolsky M. A. (2005). Antioxidant enzyme activity is up-regulated after unilateral resistance exercise training in older adults. Free Radic. Biol. Med. 39, 289–295. 10.1016/j.freeradbiomed.2005.03.024 15964520

[B42] Puigserver P. Wu Z. Park C. W. Graves R. Wright M. Spiegelman B. M. (1998). A cold-inducible coactivator of nuclear receptors linked to adaptive thermogenesis. Cell 92, 829–839. 10.1016/S0092-8674(00)81410-5 9529258

[B43] Putman C. T. Kiricsi M. Pearcey J. MacLean I. M. Bamford J. A. Murdoch G. K. (2003). AMPK activation increases uncoupling protein-3 expression and mitochondrial enzyme activities in rat muscle without fibre type transitions. J. Physiol. 551, 169–178 10.1111/j.1469-7793.2003.00169.x 12813156 PMC2343134

[B44] Qiu X. Brown K. Hirschey M. D. Verdin E. Chen D. (2010). Calorie restriction reduces oxidative stress by SIRT3-mediated SOD2 activation. Cell Metab. 12, 662–667. 10.1016/j.cmet.2010.11.015 21109198

[B45] Rardin M. J. Newman J. C. Held J. M. Cusack M. P. Sorensen D. J. Li B.. (2013). Label-free quantitative proteomics of the lysine acetylome in mitochondria identifies substrates of SIRT3 in metabolic pathways. Proc. Natl. Acad. Sci. U.S.A. 110, 6601–6606. 10.1073/pnas.1302961110 23576753 PMC3631688

[B46] Reid M. B. (2001). Invited review: redox modulation of skeletal muscle contraction: what we know and what we don't. J. Appl. Physiol. 90, 724–731. 11160074 10.1152/jappl.2001.90.2.724

[B47] Ringholm S. Olesen J. Pedersen J. T. Brandt C. T. Halling J. F. Hellsten Y.. (2013). Effect of lifelong resveratrol supplementation and exercise training on skeletal muscle oxidative capacity in aging mice; impact of PGC-1α. Exp. Gerontol. 48, 1311–1318. 10.1016/j.exger.2013.08.012 23994519

[B48] Röckl K. S. C. Hirshman M. F. Brandauer J. Fujii N. Witters L. A. Goodyear L. J. (2007). Skeletal muscle adaptation to exercise training: AMP-activated protein kinase mediates muscle fiber type shift. Diabetes 56, 2062–2069. 10.2337/db07-0255 17513699

[B49] Schwer B. Bunkenborg J. Verdin R. O. Andersen J. S. Verdin E. (2006). Reversible lysine acetylation controls the activity of the mitochondrial enzyme acetyl-CoA synthetase 2. Proc. Natl. Acad. Sci. U.S.A. 103, 10224–10229. 10.1073/pnas.0603968103 16788062 PMC1502439

[B50] Schwer B. North B. J. Frye R. A. Ott M. Verdin E. (2002). The human silent information regulator (Sir)2 homologue hSIRT3 is a mitochondrial nicotinamide adenine dinucleotide-dependent deacetylase. J. Cell Biol. 158, 647–657 10.1083/jcb.200205057 12186850 PMC2174009

[B51] Shinmura K. Tamaki K. Saito K. Nakano Y. Tobe T. Bolli R. (2007). Cardioprotective effects of short-term caloric restriction are mediated by adiponectin via activation of AMP-activated protein kinase. Circulation 116, 2809–2817. 10.1161/CIRCULATIONAHA.107.725697 18040027

[B52] Someya S. Yu W. Hallows W. C. Xu J. Vann J. M. Leeuwenburgh C.. (2010). Sirt3 mediates reduction of oxidative damage and prevention of age-related hearing loss under caloric restriction. Cell 143, 802–812. 10.1016/j.cell.2010.10.002 21094524 PMC3018849

[B53] Spina R. J. Chi M. M. Hopkins M. G. Nemeth P. M. Lowry O. H. Holloszy J. O. (1996). Mitochondrial enzymes increase in muscle in response to 7-10 days of cycle exercise. J Appl Physiol 80, 2250–2254. 8806937 10.1152/jappl.1996.80.6.2250

[B54] Tanner C. B. Madsen S. R. Hallowell D. M. Goring D. M. J. Moore T. M. Hardman S. E.. (2013). Mitochondrial and performance adaptations to exercise training in mice lacking skeletal muscle LKB1. Am. J. Physiol. Endocrinol. Metab. 305, E1018–E1029. 10.1152/ajpendo.00227.2013 23982155 PMC3798697

[B55] Tao R. Coleman M. C. Pennington J. D. Ozden O. Park S.-H. Jiang H.. (2010). Sirt3-mediated deacetylation of evolutionarily conserved lysine 122 regulates MnSOD activity in response to stress. Mol. Cell 40, 893–904. 10.1016/j.molcel.2010.12.013 21172655 PMC3266626

[B56] Tiidus P. M. Pushkarenko J. Houston M. E. (1996). Lack of antioxidant adaptation to short-term aerobic training in human muscle. Am. J. Physiol. Regul. Integr. Comp. Physiol. 271, R832–R836. 8897971 10.1152/ajpregu.1996.271.4.R832

[B57] Tonkonogi M. Walsh B. Svensson M. Sahlin K. (2000). Mitochondrial function and antioxidative defence in human muscle: effects of endurance training and oxidative stress. J. Physiol. 528, 379–388. 10.1111/j.1469-7793.2000.00379.x 11034627 PMC2270128

[B58] Treebak J. T. Pehmøller C. Kristensen J. M. Kjøbsted R. Birk J. B. Schjerling P.. (2014). Acute exercise and physiological insulin induce distinct phosphorylation signatures on TBC1D1 and TBC1D4 proteins in human skeletal muscle. J. Physiol. 592, 351–375. 10.1113/jphysiol.2013.266338 24247980 PMC3922499

[B59] Tseng A. H. H. Shieh S.-S. Wang D. L. (2013). SIRT3 deacetylates FOXO3 to protect mitochondria against oxidative damage. Free Radic. Biol. Med. 63, 222–234. 10.1016/j.freeradbiomed.2013.05.002 23665396

[B60] Vassilopoulos A. Pennington J. D. Andresson T. Rees D. M. Bosley A. D. Fearnley I. M.. (2014). SIRT3 deacetylates ATP synthase F1 complex proteins in response to nutrient- and exercise-induced stress. Antioxid. Redox Signal. 21, 551–564. 10.1089/ars.2013.5420 24252090 PMC4085980

[B61] White A. T. Schenk S. (2012). NAD+/NADH and skeletal muscle mitochondrial adaptations to exercise. Am. J. Physiol. Endocrinol. Metab. 303, E308–E321. 10.1152/ajpendo.00054.2012 22436696 PMC3423123

[B62] Wu Y.-T. Lee H.-C. Liao C.-C. Wei Y.-H. (2013). Regulation of mitochondrial F(o)F(1)ATPase activity by Sirt3-catalyzed deacetylation and its deficiency in human cells harboring 4977bp deletion of mitochondrial DNA. Biochim. Biophys. Acta 1832, 216–227. 10.1016/j.bbadis.2012.10.002 23046812

[B63] Yang Y. Cimen H. Han M.-J. Shi T. Deng J.-H. Koc H.. (2010). NAD+-dependent deacetylase SIRT3 regulates mitochondrial protein synthesis by deacetylation of the ribosomal protein MRPL10. J. Biol. Chem. 285, 7417–7429. 10.1074/jbc.M109.053421 20042612 PMC2844190

